# CREATION AND VALIDATION OF A POLYSOCIAL SCORE FOR PHYSICAL FRAILTY AMONG COMMUNITY-DWELLING OLDER ADULTS IN THE US

**DOI:** 10.1093/geroni/igac059.1299

**Published:** 2022-12-20

**Authors:** Chenkai Wu, Junhan Tang

**Affiliations:** Duke Kunshan University, Kunshan, Jiangsu, China (People's Republic); Duke Kunshan University, Kunshan, Jiangsu, China (People's Republic)

## Abstract

The interrelatedness between social determinants of health impedes researchers from identifying important social factors for health investment. A new approach is needed to quantify the aggregate effect of social factors and develop person-centered social interventions. Data are from the Health and Retirement Study; 6,075 older adults initially not frail (non-frail or prefrail) were included. Frailty was assessed by slowness, weakness, exhaustion, inactivity, and shrinking. Persons were classified as “nonfrail” (0 criteria), “prefrail” (1–2 criteria), or “frail” (3–5 criteria). We included 24 social factors from five categories (economic stability, neighborhood environment, education, community/social context, and healthcare system) and used forward stepwise regression to screen for important ones. Polysocial score was created using 15 social factors and was classified as low (0-29), intermediate (30-42), and high (43+). We used the Poisson regression to estimate the risk of incident frailty by three polysocial score categories. We found that 444 (34.5%), 651 (17.9%), and 108 (9.4%) cases of incident frailty at the 4-year follow-up among participants with a low, intermediate, and high polysocial score, respectively. In the multivariable-adjusted Poisson model, the risk of frailty among participants in the intermediate and high polysocial score categories was 35% and 59% lower than those in the low polysocial score category, respectively. We found a universal association between polysocial scores and frailty across race/ethnicity and sex subgroups. The polysocial approach may offer possible solutions to monitor social environments and suggestions for older people to improve their social status for specific health outcomes.

